# Pilot study of the use of ezetimibe in dogs with hyperlipidemia

**DOI:** 10.1007/s11259-026-11286-1

**Published:** 2026-05-27

**Authors:** Péter de Lima Wachholz, Eduarda Santos Bierhals, Camila Moura de Lima, Caroline Xavier Grala, Vitória Ramos de Freitas, Sérgio Jorge, Fábio Raphael Pascoti Bruhn, Márcia de Oliveira Nobre, Mariana Cristina Hoeppner Rondelli

**Affiliations:** Department of Veterinary Medicine, Federal University of Pelotas, Pelotas, Rio Grande do Sul Brazil

**Keywords:** Canine, Dyslipidemia, Hypercholesterolemia, Lipid disorders

## Abstract

The aim of this study was to evaluate the tolerance and efficacy of ezetimibe in reducing cholesterol and/or triglycerides in dogs with hyperlipidemia. Twenty-three dogs with hyperlipidemia were randomly distributed into three groups: group D (*n* = 7) received low-fat dietary treatment, group E (*n* = 9) received ezetimibe treatment, and group DE (*n* = 7) received low-fat dietary treatment associated with ezetimibe. Serum cholesterol, triglycerides and glucose were evaluated before (T0), between 12 and 18 days (T1) and after 30 days (T2) of treatment. The groups that received ezetimibe associated or not with dietary treatment had a significant reduction (*p* < 0.05) of serum cholesterol by the end of the study, and the group that received only dietary treatment did not show a significant reduction in cholesterolemia. None of the groups presented a significant reduction in triglycerides. The dogs’ owners did not report clinical changes with the use of the medication. The results allowed us to conclude that ezetimibe was well tolerated and effective in reducing serum cholesterol in dogs with hyperlipidemia, without adverse effects under the conditions of the study.

## Introduction

Hyperlipidemia is the increase in serum lipid concentration that can occur due to greater levels of cholesterol, triglycerides, or both. The increased serum or plasma triglycerides is called hypertriglyceridemia, while in cholesterol is referred to as hypercholesterolemia (Catanozi 2015). Dyslipidemia is a broader term used to characterize any disturbance in the quality and/or quantity of lipids and/or blood lipoproteins, which are responsible for lipid transportation in the circulation (Xenoulis 2017).

Hyperlipidemias can be primary or secondary to diseases or medications, mainly endocrine disorders, liver diseases, and nephrotic syndrome, so the main cause should be identified and, if possible, controlled. Diet is the fundamental approach for treating hyperlipidemia, and those containing less than 20% fat in metabolizable energy are recommended (Xenoulis et al. 2020). Hypolipidemic drugs are indicated in a second instance when a low-fat diet is not sufficient to correct hyperlipidemia, especially in cases of associated comorbidities (Xenoulis and Steiner 2015). The choice of medication should be based on the cause of the lipid imbalance. The most prescribed hypolipidemic medications are fibric acid derivatives (bezafibrate, fenofibrate) that decrease plasma triglyceride concentrations by stimulating lipoprotein lipase enzyme (De Marco et al. 2017), and omega-3 supplementation (De Albuquerque et al. 2021), which, although it has limited hypolipidemic effects, is an option with few adverse effects that can be associated when the response to the initial dietary approach is inadequate or limited. However, these drugs mainly act on triglyceride metabolism with little effect on cholesterol (Xenoulis and Steiner 2010).

Ezetimibe is a selective inhibitor of cholesterol absorption widely used in medicine in combination with statins due to its efficacy and lower occurrence of adverse effects (Vavlukis and Vavlukis 2018). Based on its mechanism of action, as monotherapy, it is expected that ezetimibe will reduce the intestinal absorption of cholesterol and consequently the serum concentration of chylomicrons that transport cholesterol (Araújo et al. 2005). In humans, this drug is rapidly taken up by intestinal cells and undergoes glucuronidation, forming the glucuronide form of ezetimibe. The glucuronide form is absorbed, subsequently taken up by the liver, excreted in bile, and remains on the villous border of enterocytes, blocking cholesterol absorption (Yamashita et al. 2015). Studies in mice, rats, and dogs have shown that using ezetimibe in combination with other hypolipidemic drugs is safe and acts complementarily through different mechanisms of action (Davis et al. 2001; Bays 2002). A retrospective case study on the combination of this drug with bezafibrate in the treatment of three dogs with hyperlipidemias secondary to diabetes mellitus and/or hypothyroidism showed significant reductions in triglyceride and cholesterol concentrations, with doses of ezetimibe ranging from 0.15 to 0.46 mg/kg orally every 24 h for 30 days (Wachholz et al. 2022). In a more recent study (Oda et al. 2024), ezetimibe was used as monotherapy in nine dogs diagnosed with hypercholesterolemia and undergoing treatment for endocrinopathies (Cushing’s syndrome, hypothyroidism, and/or diabetes mellitus), a reduction in cholesterol levels was observed between 2 and 4 months after the initiation of therapy.

Considering the limited effect on hypercholesterolemia of available drugs for canine hyperlipidemia (De Marco et al. 2017), ezetimibe may be an interesting alternative. Thus, the aim of this study was to evaluate the therapeutic effectiveness and tolerance of ezetimibe in reducing serum total cholesterol and triglycerides in dogs with primary or secondary hyperlipidemia, presenting hypercholesterolemia and/or hypertriglyceridemia.

## Materials and methods

### Animals and experimental group composition

The proposed experimental protocol was approved by the local Animal Experimentation Ethics Committee under number 23110.034021/2022-09. The study was conducted at the Veterinary Teaching Hospital of the Universidade Federal de Pelotas (HCV - UFPel) from January to December 2023. The minimum sample size was defined as seven animals per group using PS Power and Sample Size^®^ software, considering an 80% statistical power and a 5% significance level, based on the standard deviation of cholesterol values in dogs treated with the hypolipidemic agent bezafibrate as reported by De Marco et al. (2017)

As inclusion criteria, there were no restrictions regarding age, sex, breed, or weight. Animals with body condition scores corresponding to thin or very thin, hypertensive, with diabetes mellitus, pancreatitis, neoplasms, or undergoing treatment with corticosteroids, antiepileptics, and those ones whose primary disease treatment needed to be immediate and that might have interfered with the development of this study were not included. Dogs diagnosed with secondary hyperlipidemia did not receive treatment for the underlying cause during the study period, undergoing exclusively the experimental protocol.

During the first appointment, anamnesis was performed, including verification of the type and quantity of food provided (commercial or homemade), medications used, medical history, and overall patient condition. Dogs underwent a physical examination that included observation of mucous membranes, hydration, palpation of lymph nodes, abdominal palpation, thoracic auscultation, rectal temperature measurement, heart rate, respiratory rate, and assessment of pulse and systemic blood pressure using non-invasive Doppler vascular equipment (MEDMEGA^®^^,^ MEDMEGA Medical Equipment Industry Ltd.) according to the American College of Veterinary Internal Medicine guidelines (ACVIM) (Acierno et al. 2018).

Dogs with hyperlipidemia due to hypercholesterolemia and/or hypertriglyceridemia, with serum cholesterol levels greater than 270 mg/dL and/or serum triglyceride concentration greater than 112 mg/dL, as referenced by the Clinical Pathology Laboratory of HCV - UFPel, and the primary cause of hyperlipidemia treatment could be postponed for the experimental period of 30 days, were included.

After animal inclusion in the study, the administration method of medication and/or diet was explained to the owners. During treatment, the owner was questioned about the acceptance of the new food, adverse effects related to food and/or ezetimibe onset (such as vomiting, abdominal pain, diarrhea, apathy), and any difficulties related to treatment. Twenty-three dogs with hyperlipidemia were randomly assigned following the order of arrival into three groups, as described below.

### Group D - dietary treatment (*n* = 7)

The dogs in this group received commercial food with low-fat content (ether extract content of 5 to 9%) as examples, normocaloric fat-restricted food for dogs with ideal weight, or commercial food with fat restriction and hypocaloric content for overweight and obese dogs. The veterinarian prescribed commercial options for these foods, and the owner acquired one from the list, maintaining the use of the same food throughout the experimental period. The calculation of the food quantity was based on the nutrition and weight management guide for dogs and cats from the American Animal Hospital Association (Cline et al. 2021). Gradual introduction of the new food over one week was recommended, and the treatment lasted for 30 days, starting from the complete replacement with the new food. Six animals received a hypocaloric commercial diet consisting of 22-35.5% crude protein, 7–9% ether extract, 7.8–15% crude fiber, and 2.670–2.979 kcal/kg of metabolizable energy, and one animal received a normocaloric low-fat commercial diet with 20% crude protein, 5% ether extract, 4.3% crude fiber, and 3.431 kcal/kg of metabolizable energy.

### Group E - ezetimibe treatment (*n* = 9)

All dogs in this group were treated exclusively with ezetimibe (Zetia^®^ 10 mg; Schering-Plough, São Paulo-SP, Brazil) at an average dose of 0.38 ± 0.16 mg/kg (range, 0.23–0.70 mg/kg), postprandial, orally every 24 h. Since there is no well-defined ezetimibe dose for dogs, we extrapolated the human dose based on the dog’s body weight as follows: dogs with body weight between 1 and 10 kg received 2.5 mg (1/4 of a tablet); dogs between 10.1 and 25 kg received 5 mg (1/2 tablet); and dogs weighing between 25.1 kg and 35 kg received 10 mg (1 tablet), after one of the meals, for 30 days. Owners administered the medication at home and were instructed not to change the quantity or type of diet during the treatment period. Eight animals received maintenance commercial food with ether extract content between 15% and 18% at the beginning of the experiment, including super premium (*n* = 7) and premium (*n* = 1) types, while one dog received homemade food.

### Group DE - dietary treatment and ezetimibe administration (*n* = 7)

All dogs in this group received commercial low-fat food (ether extract content of 5 to 9%) associated with ezetimibe administration at an average dose of 0.46 ± 0.19 mg/kg (range, 0.33–0.89 mg/kg), postprandial, orally every 24 h after one of the main meals for 30 days. The dose was extrapolated from human use based on the dog’s body weight, as previously mentioned. Five animals received a hypocaloric commercial food consisting of 22-35.5% crude protein, 7–9% ether extract, 7.8–15% crude fiber, and 2.670–2.979 kcal/kg of metabolizable energy, and two received a normocaloric low-fat commercial diet with 20% crude protein, 5% ether extract, 4.3% crude fiber, and 3.431 kcal/kg of metabolizable energy.

The respective owners were responsible for both the dietary and ezetimibe treatment, and should follow all the given instructions. During the appointments, they were instructed on how to administer the medication and the appropriate food quantity. Additionally, they were advised to discontinue providing treats to individuals in groups D and DE. The medication used was from the same pharmaceutical specialty and was provided by the researchers.

### Biochemical evaluations

Serum concentrations of total cholesterol, triglycerides and blood glucose were evaluated before treatment (T0), between 12 and 18 days of treatment (T1), and at the end of treatment (T2). In group D, two patients were evaluated at 60 days of treatment, while the others were assessed between 30 and 32 days, according to the owners availability. All dogs in groups E and DE received ezetimibe for 30 days and were evaluated 31 days after the treatment onset (ranging from 30 to 32 days).

Blood samples were collected after a solid fast of 8 to 12 h, via jugular venipuncture, cranial cephalic vein, or saphenous vein using a 20 × 0.55 mm needle or 23G scalp. Samples were deposited in clot activator tubes, centrifuged. Serum samples were processed at the Veterinary Clinical Pathology Laboratory of the HCV - UFPel. Measurements of total cholesterol and triglycerides were performed using the enzymatic colorimetric method, and serum glucose was measured enzymatically with hexokinase or using a validated portable glucometer for dogs (Freestyle Optium Neo^®^; Santos et al. 2022).

### Weight and body condition score assessment

Body weight was assessed in all three timepoints, and body condition score was established by physical examination at T0 and T2, based on a scale of one to nine according to the system described by Laflamme (1997). Lean body mass score was determined using a four-point scale (0 = severe muscle loss, 1 = moderate muscle loss, 2 = mild muscle loss, 3 = normal muscle mass) for nutritional assessment in dogs, according to the World Small Animal Veterinary Association (WSAVA) nutrition tool (WSAVA Global Nutrition Committee 2011).

### Investigation of hyperlipidemia etiology

In order to investigate the cause of hyperlipidemia, anamnesis was conducted with the dog owners. Physical examination, and an initial laboratory screening were performed in all dogs, including complete blood count, measurement of liver enzymes (alanine aminotransferase, alkaline phosphatase), renal markers (creatinine and urea), plasma protein assessment, venous blood gas analysis, and urinalysis. Basic imaging tests, such as abdominal and/or cervical ultrasound, were also conducted. Subsequently, the most appropriate specific tests were recommended based on the patient’s history, physical examination, and additional tests. These specific tests included measuring serum cortisol after low-dose dexamethasone suppression tests, and serum thyroid-stimulating hormone (TSH) levels, total serum thyroxine (T4), free serum thyroxine (T4) by equilibrium dialysis, to rule out hypercortisolism and hypothyroidism, respectively.

### Statistical analysis

The results related to cholesterol levels, triglyceride levels, blood glucose, weight, body condition score, and ezetimibe dosage were recorded in a table, and the arithmetic mean, standard deviation, and range (minimum and maximum values) were calculated. Statistical analysis of the data was performed using IBM SPSS Statistics 20^®^. The studied data had normal distribution verified by the Shapiro-Wilk test; therefore, parametric tests were used. For serum cholesterol, serum triglycerides, blood glucose, and weight variables, repeated measures analysis of variance (ANOVA) was conducted, and the variation in ezetimibe dosage was compared also using ANOVA. The BCS variable was analyzed using non-parametric Kruskal-Wallis and Friedman tests. The significance level considered was *p* < 0.05.

## Results

For the composition of the study groups, 59 dogs were seen at the endocrinology service of the Veterinary Teaching Hospital of the Universidade Federal de Pelotas. Of those, 26 were included due to hyperlipidemia. Two animals from group D and one dog from group DE were excluded due to owners’ option in quitting the research, resulting in a total of 23 dogs. The unequal group sizes resulted from owner-related exclusions, which are inherent to clinical studies involving privately owned animals. Initial recruitment exceeded the minimum calculated sample size to account for potential losses. Exclusions occurred randomly and were unrelated to experimental criteria. Importantly, all groups maintained a sample size equal to or greater than the minimum required for statistical validity. Table 1 provides details on sex, age, breed, weight, body condition score, lean body mass score, and the dose of ezetimibe used.

**Table 1:** Sex, neutered/spayed, age, breed, body weight, body condition score (BCS), and lean mass score (LMS) of dogs with hyperlipidemia in groups D (dietary treatment), E (treatment with ezetimiba), and DE (dietary treatment combined with ezetimibe administration), and individual ezetimibe doses (mg/kg) in groups E and DE

Group	Dog	Sex	Neutered/Spayed	Age (Years)	Breed	Body Weight (Kg)	BCS	LMS	Ezetimibe dose (mg/kg)^†^
D	1	Female	Yes	14	Yorkshire Terrier	3.6	7	3	N/A
	2	Female	Yes	5	Yorkshire Terrier	4.7	4	3	N/A
	3	Female	Yes	5	Shih-tzu	7.7	6	3	N/A
	4	Female	Yes	3	Shih-tzu	7.2	7	3	N/A
	5	Female	Yes	11	MBD	34.5	7	2	N/A
	6	Female	Yes	9	Dachshund	9.95	8	2	N/A
	7	Male	Yes	9	Maltese	3.5	5	3	N/A
Mean ± SD	-----			8±3.8		10.16 ± 10.99			N/A
E	1	Female	Yes	8	MBD	19	8	3	0.26
	2	Female	Yes	16	MBD	20.8	7	2	0.24
	3	Female	Yes	16	Miniature Pinscher	4.25	4	1	0.59
	4	Female	Yes	13	Maltese	5.2	8	1	0.48
	5	Male	No	4	Whippet	15.5	5	3	0.32
	6	Male	Yes	9	Pinscher	3.55	6	2	0.7
	7	Female	Yes	9	Shih-tzu	11	6	3	0.23
	8	Male	Yes	8	MBD	34.6	8	3	0.29
	9	Female	Yes	7	Miniature Schnauzer	7.8	6	3	0.32
Mean ± SD	-----			10±4,1		13.52 ± 10.15			0.38 ± 0.16
DE	1	Female	Yes	11	MBD	30	7	3	0.33
	2	Female	Yes	9	Pinscher	2.8	7	3	0.89
	3	Female	Yes	10	MBD	25.5	7	3	0.39
	4	Female	Yes	6	Shih-tzu	10.5	6	3	0.48
	5	Female	Yes	10	MBD	13.6	8	3	0.37
	6	Female	Yes	9	Shih-tzu	11.25	8	3	0.44
	7	Female	Yes	7	Miniature Schnauzer	7.3	5	3	0.34
Mean ± SD	-----			9±1.7		14.42 ± 9.81			0.46 ± 0.19

^†^Every 24 hours, for 30 days. *N/A* not applicable. *SD* Standard deviation. *MBD* Mixed breed dog

### Serum cholesterol concentration

Mean serum cholesterol levels of groups D, E, and DE were higher than the maximum reference value at the initial assessment, and by the end of the study, only groups E and DE presented mean values within the reference range (Table 2).

**Table 2:** Mean serum concentrations of total cholesterol and triglycerides in dogs with hyperlipidemia submitted to dietary treatment (group D), ezetimibe treatment (group E) or dietary treatment associated with ezetimibe administration (group DE), at three timepoints of the study

Indicators	Group	T0	T1	T2
Cholesterol (mg/dL) mean ± standard deviation (range)	D	433 ± 175^a^	247 ± 62^b^	327 ± 105^a^
		(264 - 752)	(191 - 314)	(208 - 479)
	E	365 ± 162^a^	246 ± 121^b^	126 ± 140^b^
		(229 - 753)	(125 - 441)	(140 - 537)
	DE	342 ± 99^a^	224 ± 131^b^	228 ± 115^b^
		(221 - 447)	(109 - 377)	(110 - 381)
Cholesterol reference values (mg/dL)		135 - 270	135 – 270	135 - 270
Triglycerides (mg/dL) mean ± standard deviation(range)	D	272 ± 301	114 ± 37	228 ± 248
		(56 - 737)	(74 - 148)	(59 - 730)
	E	476 ± 278	318 ± 283	409 ± 507
		(100 - 986)	(57 - 928)	(49 - 1383)
	DE	912 ± 569	296 ± 433	371 ± 362
		(70 - 1832)	(70 - 1071)	(64 - 927)
Triglycerides reference values (mg/dL)		20 – 112	20 – 112	20 – 112

Different lowercase letters in the same line indicate significant difference between timepoints (*p* < 0.05)

**Fig. 1:**
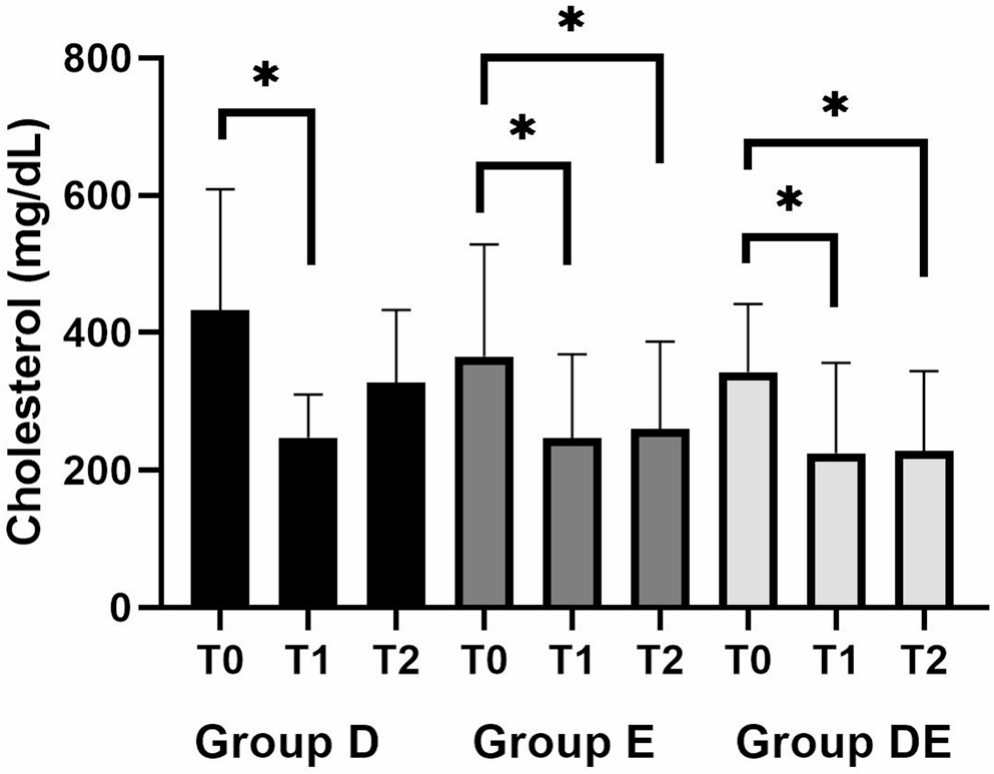
Graphical representation of mean serum total cholesterol concentrations in dogs with hyperlipidemia undergoing dietary treatment (group D), ezetimibe treatment (group E), or dietary treatment combined with ezetimibe administration (group DE) at three study timepoints. Bars represent the mean serum cholesterol values, and lines indicate the standard deviation. *Indicates significant difference (*p* < 0.05) between time points

The group treated exclusively with a low-fat diet (group D) did not show a significant reduction in cholesterol between time points T0 and T2 (*p* = 0.755), however, there was a significant reduction between T0 and T1 (*p* = 0.018). The group receiving ezetimibe (group E) demonstrated a reduction in serum cholesterol at T1 (*p* = 0.024) and T2 (*p* = 0.001) compared to baseline (T0). Similarly, a reduction in cholesterol was observed in the group receiving dietary treatment combined with ezetimibe administration (group DE) at baseline (T0) and T1 (*p* = 0.001), as well as after 30 days of treatment (T2) (*p* = 0.016) (Fig. 1).

In group D, 85.71% (6/7) of the animals had hypercholesterolemia. Among them, 50% (3/6) had their serum concentration between reference range by the end of treatment, 33.33% (2/6) experienced a considerable reduction, and 16.66% (1/6) showed a moderate increase in serum cholesterol levels. In group E, 77.77% (7/9) of the patients had hypercholesterolemia. Among them, 42.85% (3/7) achieved reference values by the end of treatment, 42.85% (3/7) experienced a reduction, and 14.28% (1/7) had a mild increase. The other two dogs (2/9) with normal cholesterol levels at T0 showed slightly reduced values at T2. In DE group, 71.42% (5/7) of the dogs had hypercholesterolemia. Among them, 40% (2/5) reduced to normal levels, and 60% (3/5) exhibited lower values compared to the initial concentrations over the 30-day treatment period. The other two animals (2/7) with normal serum cholesterol values also showed a slight reduction after treatment.

### Serum triglyceride concentration

At the beginning of the study, mean serum triglyceride levels in groups D, E, and DE were above the reference values. Upon completion of the treatment, none of the groups exhibited values within the reference range. In group D, 57.14% (4/7) of the animals had hypertriglyceridemia, and by the end of treatment, 42.85% (3/7) showed a reduction in triglyceride levels. In group E, hypertriglyceridemia was observed in 88.88% (8/9) of dogs throughout the study, with 55.55% (5/9) experiencing a decrease in triglyceride levels and 33.33% (3/9) showing an increase. Finally, in group DE, 85.71% (6/7) had hypertriglyceridemia, and 71.42% (5/7) exhibited a decrease in serum triglyceride levels. However, there was no significant difference (*p* > 0.05) in triglyceride reduction among any of the proposed treatments.

### Blood glucose concentration

In group D, mean (± SD) blood glucose level was 90.13 mg/dL (± 8.78) before treatment (T0) and 105.44 mg/dL (± 13.84) after treatment (T2). For group E, the mean (± SD) was 101.05 mg/dL (8.87) at the start, decreasing to 96.44 mg/dL (± 15.89) after 30 days of treatment. In group DE, mean (± SD) was 95.97 mg/dL (± 10.49) at T0 and 95.77 mg/dL (± 12.15) after treatment. There was no significant difference in blood glucose levels between the groups (*p* = 0.238) or comparing treatment times (*p* = 0.573).

### Ezetimibe dosage

The mean dose of ezetimibe administered to the dogs was 0.42 mg/kg ± 0.18. Group E received 0.38 mg/kg ± 0.16 (range, 0.23–0.7), while group DE, 0.46 mg/kg ± 0.19 (range, 0.33–0.89). There was no significant difference (*p* = 0.297) in dose comparison between dogs and among the groups.

### Weight and body condition score (BCS)

The lowest recorded weight was 2.8 kg, and the greatest was 34.6 kg. At the initial assessment, animals in group D had an average weight (± SD) of 10.16 kg (± 10.99), group E had 13.52 kg (± 10.15), and group DE, 14.42 kg (± 9.81). After treatment, mean weights (± SD) were 9.79 kg (± 10.48), 13.28 kg (± 10.18), and 14.06 kg (± 9.42), in groups D, E and DE, respectively. Despite a statistically significant difference in weight (*p* = 0.014) between timepoints, there was no significant difference in body condition score (BCS) (*p* > 0.05) between the beginning and end of the experiment in groups D and DE, while mild weight loss occurred in four animals from group D and three from group DE. The mean reduction in body weight was 4.81% (± 1.2) for both groups D and DE, with the lowest loss being 2.01% and the highest 6% of body weight. In group E, there was no significant change in these parameters.

### Underlying causes of hyperlipidemia

The primary diagnosis established for dogs presenting with hyperlipidemia was primary hypothyroidism in 13.04% (*n* = 3). For most individuals (73.91%), the cause remained undefined. Table 3 displays the defined and undetermined causes of hyperlipidemia for the dogs included in this study.

**Table 3:** Underlying causes of hyperlipidemia in the dogs of this investigation

Etiology of hyperlipidemia in dogs	*n* (%)
Primary hyperlipidemia	1 (4.34)
Primary hypothyroidism	3 (13.04)
Hypercortisolism	1 (4.34)
Chronic glucocorticoid replacement therapy in primary hypoadrenocorticism	1 (4.34)
Undetermined cause of hyperlipidemia*	17 (73.91)

*secondary causes for hyperlipidemia were not found following clinical investigation. In this category, dogs that tested negative for hypercortisolism and had normal thyroid hormonal screening were included, as well as those whose owners have not allowed further investigation

## Discussion

In groups E and DE groups, a reduction in serum cholesterol levels was observed in dogs either with hypercholesterolemia or those with normal cholesterol values (they were included in the study for having hypertriglyceridemia). It was observed that after 12 days of treatment with ezetimibe, whether associated with a low-fat diet or not, there was a decrease in this variable. Other reports have also shown that the reduction in serum cholesterol in dogs treated with ezetimibe occurred along with other drugs, such as simvastatin, atorvastatin, pravastatin, lovastatin (Davis et al. 2001) or bezafibrate (Wachholz et al. 2022).

Dogs that received only dietary treatment (group D) did not present significant reduction in cholesterol after 30 days of treatment. In contrast, Miceli et al. (2021) observed that dogs with hypercholesterolemia returned to normal serum levels after one month of exclusive dietary treatment with a low-fat diet in 50% of cases. Xenoulis et al. (2020) also observed a reduction in cholesterol below reference values in 37.5% of Schnauzer dogs with hypercholesterolemia that received a low-fat diet for 8 weeks. In contrast, dogs in group D may not have shown a significant improvement in cholesterol levels because most of the cholesterol comes from hepatic synthesis and intestinal reabsorption of bile acids, with only a small amount originating from the diet (Pertsemildis et al. 1973; Melchior and Harwell 1985; Xenoulis 2017). Furthermore, it is possible that the cause of hyperlipidemia in these dogs was not of dietary origin, as suspected during the clinical evaluation conducted in this study.

The reduction of triglyceridemia was not expected as a main effect of treatment with ezetimibe, as its mechanism of action is related to cholesterol levels. However, 78.26% (18/23) of the dogs presented hypertriglyceridemia, and in 33.33% (6/18), results back to normal range occurred after 30 days of treatment. In the groups that received dietary treatment with low-fat food (groups D and DE), 71.42% (10/14) had hypertriglyceridemia, and 25% (4/10) presented reduction in triglyceride levels to reference values at the end of treatment. In the group where the original diet was maintained and ezetimibe was added (group E), 88.88% (8/9) exhibited hypertriglyceridemia, and 22.22% (2/8) had normal triglycerides levels at T2. Miceli et al. (2021) compared two groups of dogs diagnosed with hypertriglyceridemia treated with fenofibrate or a diet containing 10% fat for one month. Only 26.6% of the dogs undergoing dietary treatment had normal serum triglyceride levels by the end of treatment. In contrast, 85.93% of the dogs receiving fenofibrate showed improvement in hypertriglyceridemia during the same treatment period. In a study conducted by Xenoulis et al. (2020) evaluating the efficacy of a commercial low-fat diet in Schnauzer dogs with hyperlipidemia, there was a reduction in hypertriglyceridemia to values below 500 mg/dL after 8 weeks of treatment. Xenoulis and Steiner (2015) noted that while there are many commercial foods with reduced fat content, other factors can influence triglyceride reduction, such as the type of fat and fiber content. The absence of triglyceride differences may be related to the possibility of dietary treatment for hyperlipidemia is longer than four weeks or the need to indicate an ultra-low-fat homemade diet, and/or combine it with drugs for better results (Xenoulis and Steiner 2010).

In the discussed study, low-fat commercial extruded diets were prescribed, either for obesity, with 7–9% of ether extract (*n* = 6 in group D; *n* = 5 in group DE), or for gastrointestinal disorders with low-fat content (5% ether extract) (*n* = 1 in group D; *n* = 2 in group DE). The decision about the type of food was based on the dog´s body condition score: for overweight and obese dogs, obesity diets were prescribed in a list of commercial pet foods, given the diverse options available in Brazil, and the owners could choose which one to buy, according to costs, availability, and acceptance by the dog. For dogs with normal body condition (BCS 5/9), once losing weight was not the main goal, the same low-fat commercial diet was indicated for the three mentioned cases. Although the dogs received different foods, they were similar in ether extract content, thus, are indicated for hyperlipidemia (Xenoulis and Steiner 2015).

Furthermore, ezetimibe is a medication that acts on reducing the absorption of dietary cholesterol by binding to the Niemann-Pick C1-Like (NPC1L1) receptors located in enterocytes. It does not interfere with the absorption of dietary triglycerides. However, it is believed that reducing the delivery of exogenous cholesterol may decrease the synthesis and secretion of VLDL by the liver, leading to reduced serum triglyceride levels (Yamashita et al. 2015). We decided to include dogs with hypertriglyceridemia, with or without hypercholesterolemia, because dyslipidemia typically occurs as a mixed clinical condition. Similarly, De Marco et al. (2017) and Miceli et al. (2021) also evaluated the efficacy of proposed treatments on both lipids, although the primary goal of these studies was to assess the effectiveness in reducing triglyceride levels in the studied dogs.

Glucose evaluation was performed to observe whether there would be a reduction with hyperlipidemia treatment. Insulin resistance due to hypertriglyceridemia is described in humans, but few studies have assessed the relationship between hyperlipidemia and insulin resistance in dogs. Xenoulis et al. (2011) compared serum insulin, glucose, and triglyceride concentrations in Schnauzer dogs diagnosed with primary hypertriglyceridemia to healthy dogs of the same breed. They observed that mean serum insulin concentration was significantly higher in the primary hypertriglyceridemia group, although there was no difference in blood glucose levels between the groups. In our study, similarly to what the cited authors have reported, there was no significant difference in blood glucose levels between the groups over time. However, serum insulin levels were not measured, so, no conclusions can be made about the occurrence of insulin resistance.

For humans, the recommended dose of ezetimibe is 10 mg/day, administered on fasting or postprandial (Lipka 2003). Since there is no established dose for veterinary medicine, the dosage in this study was extrapolated from human medical indications. The use of ezetimibe in dogs was initially reported by Davis et al. (2001) in a study where ezetimibe was administered at a dose of 0.007 mg/kg/day, either alone or in combination with lovastatin (5 mg/kg/day), to dogs on a cholesterol-rich diet. Although neither medication alone significantly reduced cholesterol levels, the combination therapy resulted in a 50% reduction in cholesterol levels after 14 days of treatment. In order to standardize dosing and due to the availability of only a 10 mg commercial formulation in tablets, ezetimibe doses were distributed based on three weight categories and subsequently compared between groups (groups E and DE). Despite variations in the dose used among dogs, there was no significant difference in this analysis, indicating similar dose across the study animals (0.38 and 0.46 mg/kg/day for 30 days). Interestingly, similar doses of ezetimibe were reported as effective in decreasing total cholesterol levels in nine dogs with secondary hypercholestolemia due to hypercortisolism. Mean doses of ezetimibe from 0.027 ± 0.010 mg/kg/day for 2 months, and 0.040 ± 0.023 mg/kg/day for 4 months were reported, although the dose increase did not influence cholesterol levels over time (Oda et al. 2024). Another consideration is that tablet splitting was necessary to adjust the medication dose, which may have affected efficacy in dogs receiving split tablets compared to those receiving whole tablets. However, only two dogs received whole tablets, which seemingly has not influenced the results.

None of the patients undergoing pharmacological treatment exhibited adverse effects during the experimental period or thereafter, such as nausea, vomiting, and/or diarrhea. In humans, the use of ezetimibe is safe and has not shown significant adverse effects in clinical screenings, where individuals receiving the medication and placebo reported the same clinical signs, such as abdominal pain, diarrhea, or flatulence (Kosoglou et al. 2005; Yamashita et al. 2015).

It is important to note that animals in groups D and DE that experienced weight loss exhibited a mean reduction of only 4,81% from the onset to the end of treatment. Machado and Jericó (2015) reported that a weight reduction of 7 to 10% from baseline is necessary for dogs with obesity-associated comorbidities to exhibit improvements in altered clinical assessments. Therefore, it is unlikely that weight loss had an impact on the variables of triglyceridemia and cholesterolemia. The etiology of hyperlipidemia was successfully determined in 26.08% (6/23) of the animals. Among the undefined causes, 34.78% (8/23) of the dogs exhibited clinical findings suggestive of hypercortisolism. However, four dogs had negative results on the low-dose dexamethasone suppression test, and four owners opted not to pursue further investigation. One patient presented with serum TSH levels at the upper limit of the reference range and total T4 and free T4 levels at the lower limits of reference range on thyroid screening. However, these findings were not conclusive for hypothyroidism. Eight dogs have not presented clinical signs or complementary tests suggestive of primary diseases; therefore, specific tests were not indicated. Some authors suggest that hypercortisolism can cause hyperlipidemia in the absence of other clinical signs (Fleeman and Barret 2023). However, ALIVE (Agreeing Language In Veterinary Endocrinology) project discourages the use of specific tests such as low-dose dexamethasone suppression and/or ACTH stimulation in dogs without clinical signs for diagnosing hypercortisolism (ALIVE 2024; Niessen et al. 2025; Niessen et al. 2026).

In a retrospective study conducted by Usui et al. (2015), the influence of fasting time on the assessment of serum cholesterol, triglycerides, and lipoproteins in dogs was evaluated. The authors reported no significant differences between animals fasted for 8 to 12 h and those fasted for more than 12 h. Interestingly, dogs fasted for over 12 h exhibited higher mean serum cholesterol levels (289.9 ± 1.02 mg/dL) compared to those fasted for less than 8 h (279.1 ± 1.03 mg/dL). These findings support the adequacy of the 8-to-12-hour fasting protocol adopted in the present study for lipid profile evaluation.

Some limitations of this study are related to the administration of ezetimibe and food by the owners at home. Regarding the diets of groups D and DE, owners selected the dietary treatment from the options on the prescription list, and in group E, there was no standardization of the diet. Due to the nature of the clinical study, some owners did not attend the scheduled appointments, and therefore some blood analysis were performed before or after 15 days of treatment. In two cases of group D, recheck was performed 60 days after treatment onset. Also, the short follow-up period (30 days) may not have allowed us to consider long-term adverse effects of this medication use; therefore, safety should be evaluated in more details for chronic use.

## Conclusions

Ezetimibe has shown to be a promise for treating dogs with hypercholesterolemia. When used in combination with or without a low-fat diet, it effectively reduces cholesterol levels in dogs with hyperlipidemia, regardless of the cause, compared to dietary treatment with a reduced-fat diet. Neither of the proposed treatments significantly reduced triglyceride levels. Adverse effects such as vomiting, diarrhea, abdominal pain, or apathy were not reported by the owners; however, long-term studies are necessary to ensure its safety in dogs for prolonged periods.

## Data Availability

The datasets generated and analyzed during this study are included in this article. For further inquiries, please contact the corresponding author.
